# Long-Acting Injectable Antipsychotics (LAIs) Prescribing Trends during the COVID-19 Pandemic in Romania

**DOI:** 10.3390/healthcare10071265

**Published:** 2022-07-07

**Authors:** Ana A. Miron, Petru I. Ifteni, Andreea Teodorescu, Paula S. Petric

**Affiliations:** 1 Faculty of Medicine, Transilvania University of Braşov, 500036 Brașov, Romania; petru_ifteni@yahoo.com (P.I.I.); andre_martie@yahoo.com (A.T.); paula_petric@yahoo.com (P.S.P.); 2 Spitalul Clinic de Psihiatrie și Neurologie Brașov, 500123 Brașov, Romania

**Keywords:** long-acting injectable antipsychotics, pandemic, relapse, schizophrenia

## Abstract

Long acting injectable antipsychotics (LAIs) are considered the ideal treatment for schizophrenia, especially for young patients with high rates of non-adherence. In the current COVID-19 pandemic, it has been reported that the administration of LAIs decreased in some areas. The aim of this study was to evaluate the impact of COVID-19 pandemic on the initiation of LAIs. This is a retrospective mirror- image study covering a total period of 24 months: 12 months before and 12 months after the declaration of COVID-19 pandemic on March 11, 2020. During the study period, out of 218 patients admitted with schizophrenia, only 15 (1.3%) received LAIs at discharge. There was a 48.3% reduction in LAIs initiation compared to the pre-pandemic period (29 LAIs initiations in 2019 from 224 admissions). Despite the 27% reduction in the total number of admissions (1500 in 2019 vs. 1100 in 2020), the number of admissions with schizophrenia remained almost the same (224 in 2019 vs. 218 in 2020). COVID-19 pandemic brought an important challenge in the treatment of patients with schizophrenia, especially in the initiation of LAIs. This could have an important impact on the relapse rate in the next period.

## 1. Introduction

LAIs (long-acting injectable antipsychotics) are considered the ideal treatment for schizophrenia and schizoaffective disorder for patients with variable insight which affects oral medication adherence [1]. It includes different formulations of FGA (first generation antipsychotics) such as haloperidol, zuclopenthixol, flupenthixol and fluphenazine, or SGA (second generation antipsychotics) including aripiprazole, olanzapine, risperidone, and paliperidone. LAIs are administered at regular intervals, ranging from 2 weeks to 3 months, with strong evidence in reducing relapses, hospitalizations and deaths [2,3,4].

Worldwide, changes in psychotropic prescriptions overall have suffered significant changes during the COVID-19 pandemic. A population- wide trend analysis of the psychotropic medication uptake in Northern Ireland showed strong upward trends for all medications from 2012 to 2020. In March 2020, along with imposed restrictions, psychotropics’ uptake increased beyond expected values. In April–May 2020 a decrease was registered, and a return to the expected trend. Uptake of antidepressants, antipsychotics, and antiepileptics remained as expected when stratified by gender, age, single-person household, deprivation, and urbanicity [5]. Another population- wide study from Canada showed a decrease in new prescriptions for antidepressants and anxiolytics in the 3 months following COVID-19 imposed restrictions, followed by an increase in the new use of antidepressants and antipsychotics at the end of 2020, particularly in females and people aged 40 years and older [6]. Regarding the specific use of LAIs, it has been reported that the administration of LAIs has been suspended or has decreased in some areas despite the fact that it is a necessary treatment which should be continued [7]. Although not focused on LAI initiations, a Pittsburgh-based study found a non- significant decrease in LAI administrations [2]. In Canada, the rates of LAI prescriptions remained stable for the duration of COVID-19 pandemic [8]. There are some patients for whom it is safe to switch to an oral medication, but there are others for whom this change could be destabilizing. The COVID-19 pandemic has affected the provision of psychiatric care across the world, the structure of psychiatric care services, even the training of residents [9]. For those with severe and chronic mental illness, the use of long-acting formulations can reduce mortality and utilization of emergency rooms in a pandemic [10]. Furthermore, LAI treatment can also ensure a better functioning level for schizophrenia patients, so they can practice social distancing and even ensure online interviews and prescriptions during the pandemic [11].

The aim of this study was to evaluate the initiation of LAIs before and after declaration of COVID-19 pandemic.

## 2. Materials and Methods

### 2.1. Data Source

We conducted a retrospective mirror- image study in which we analyzed the initiations of LAIs for 12 months after the declaration of the COVID-19 pandemic on 11 March 2020, respectively 12 months before the pandemic. Data was retrieved from the paper and electronic documents of the patients admitted with a diagnosis of schizophrenia in the Clinical Hospital of Psychiatry and Neurology Brasov, Romania. All patients were aged over 18 years, all diagnosed with schizophrenia; the database collected basic demographic data, drug product information (type, formulation, dosage). The hospital is an academic public setting with 150 beds for acute patients, with 3 departments and 24/7 emergency service. The study was approved by the hospital’s ethics committee and it was a part of a doctoral research. Research included data regarding all available LAI formulations in Romania.

### 2.2. Study Design

**Figure 1:**
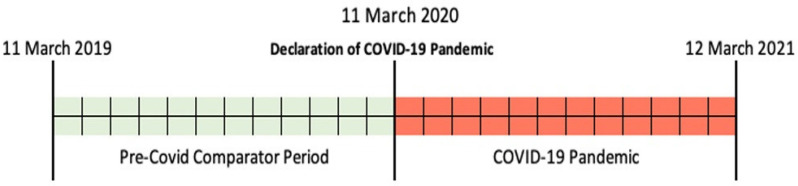
Study timeline.

Using a retrospective design, data were collected from 11 March 2019 to 12 March 2021, for all schizophrenia patients aged over 18 years that were admitted in our hospital. COVID-19 pandemic was declared by World Health Organization (WHO) on 11 March 2020. The following 12 months (12 March 2020 to 12 March 2021) were considered as the maintenance COVID period (for simplification, this period will be further referred to as “2020”), and the pre-COVID-19 comparator period was considered 11 March 2019 to 11 March 2020 (further referred to as “2019”), as shown in Figure 1.

Patients were selected according to the following inclusion criteria: age over 18, schizophrenia diagnosis according to DSM-5 criteria, first time initiated on any form of LAI. Patients already on a form of LAI treatment, and patients with a history of LAI treatment (LAI re-initiations) were excluded from the final analysis.

### 2.3. Statistical Analysis

Results were analyzed using SPSS program version 20.00. The adjusted odds ratio (AOR) with 95% CI was calculated and *p*-values less than 0.05 using *t*-test method. The multivariable logistic regression was considered to indicate a significant association.

## 3. Results

The first generation antipsychotics (FGA-LAI) captured by this dataset were haloperidol, zuclopenthixol and flupenthixol, while the second generation antypsichotics (SGA-LAI) were aripiprazole, olanzapine, risperidone, and paliperidone. To date, there are no generic formulations available in Romania for LAIs. Antipsychotics that have LAI formulations are listed in Table 1.

Between 12 March 2020 and 12 March 2021, a number of 1100 patients were hospitalized, of which 218 (19.8%) were diagnosed with schizophrenia.

**Figure 2:**
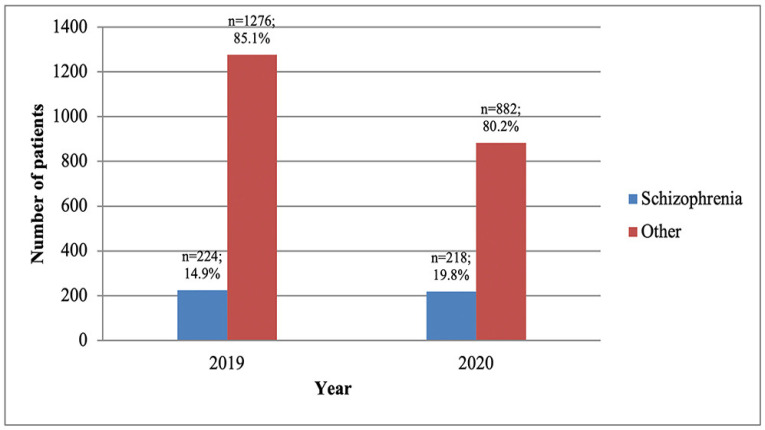
The total number of hospitalization and the number of cases with schizophrenia.

We noticed that during the pandemic the total number of hospitalizations decreased by 27% (1100 in 2020 vs. 1500 in 2019); the number of cases admitted with a diagnosis of schizophrenia was almost the same (218 vs. 224), as shown in Figure 2.

Out of the 218 patients discharged, 15 (6.9%) received LAI, 165 (75.7%) received oral antipsychotics (OAs) and 38 (17.4%) received clozapine. The comparison with the previous period is presented in Table 2.

The characteristics of the patients that were initiated on LAI treatment in 2019 and 2020 were relatively similar. Mean age in the 2019 group was 42.3 (±7.8) and 44.4 (±8.3) in 2020, respectively. We noticed that in the 2020 group, age of onset was slightly younger (25.2 (±6.6) vs. 28.6 (±8.6) in 2019), duration of illness was higher (19.2 (±6.2) vs. 13.8 (±9.2) in 2019) and length of stay was also slightly increased (16.7 (±6.6) vs. 14.1 (±8.6) in 2019).

SGA-LAIs initiation overall decreased by 48.3% after the declaration of COVID-19 pandemic and restrictions related to it. Aripiprazole LAI and Paliperidone LAI had the highest number of initiations in 2019 (n = 9); in both cases, an important decrease was registered in 2020 (n = 2). Risperidone LAI was the only one that registered a small, non- statistically significant, increase in initiations in 2020 (8 patients in 2019 vs. 11 patients in 2020; *p* = 0.2). Olanzapine LAI had the lowest number of initiations in 2019 (n = 3), and no initiation at all in 2020.

## 4. Discussion

To our knowledge, this is the first study to show data on the decrease in the initiation of LAIs during the COVID-19 pandemic, in a clinical hospital experienced in LAI treatment of schizophrenia. Although the number of admissions with schizophrenia remained at about the same level compared to the previous period (218 cases in 2020 vs. 224 cases in 2019), the number of LAIs initiations was significantly lower (15 cases in 2020 vs. 29 in 2019).

Several reasons could explain these findings. The first reason could be a lower number of hospitalizations of patients diagnosed with schizophrenia. Our results show that this actually did not happen in the pandemic period, since the number of patients admitted for relapse in schizophrenia remained relatively constant.

The second possible cause for this decrease could be the physicians’ decision not to expose the patient to contact with others during the injection procedure. Usually, the initiation of LAI was being made after long discussions with the patient and his family, which was not so easy to do during the pandemic. Correll et al., in a recent article, noticed that face-to-face consultations registered a significant decrease and LAI prescriptions were obviously affected early on in the pandemic [12]. Aside from the decrease in LAI initiation due to the social distancing measures imposed during the pandemic, another important consequence of the pandemic was that many patients already stabilized on LAIs were switched to the oral equivalents, in order to avoid contact with medical staff during regular visits for administrations, or due to suspended ambulatory services. This resulted in relapse in most cases [13].

In Romania, LAIs are usually initiated in psychiatric departments and they were administered in psychiatry hospitals, during day admissions, for better monitoring the patients. Moreover, olanzapine- LAI requires a period of minimum 3 h of medical monitoring after administration, and a family member must accompany the patient home (these measures are imposed for the prevention of post- injection delirium/sedation syndrome) [14].

During the COVID-19 pandemic, a state of emergency was declared; some of the measures imposed by the government had an obvious impact on the functioning of the health system. The hospital’s integrated ambulatory unit was temporarily suspended, as well as most of the private psychiatric practices; hospitals were only allowed to admit emergencies and day admissions were suspended. Access of patients’ family members in the hospitals was not permitted. Patients’ access in private units for administration of LAIs was difficult in terms of costs, restricted hours, and longer distances.

After that, as a result of previous restrictions, patients’ and their families’ time spent with the hospital medical staff was limited, ambulatory face-to-face consultations were limited; most prescriptions were a result of telemedicine and online/phone consultations, however a therapeutic decision of LAI initiation is challenging. We consider all these as important factors that contributed to the significant decrease noticed in the initiation of LAIs.

Moreover, LAI initiation management requires the evaluation of the tolerability of an antipsychotic initially administered orally in order to subsequently introduce the LAI form. Our results show that 18% of patients received quetiapine and amisulpride, two types of SGAs without LAI formulation. In 36.5% of cases, oral olanzapine was administered. Olanzapine LAI, which has a special administration protocol, extremely difficult to be performed in COVID-19 pandemic, had no initiation in 2020. Table 3 shows the oral antipsychotics on which the patients were stabilized.

A third reason could be that the lower initiation of LAIs is due to the restrictions imposed by COVID-19 pandemic resulting in too short hospitalizations. In our study, this impediment did not exist because the average duration of hospitalization was even longer in 2020 compared to 2019 (16.7 ± 6.6 vs. 14.1 ± 8.6, *p* < 0.001).

Another possible reason could be cost- related. SGA-LAIs are much more expensive than their oral equivalents. However, in Romania, LAIs prices are entirely supported by the public insurance system for the schizophrenia patients and over 90% of schizophrenia patients have some form of public insurance (disability assistance, disability pension, etc); therefore in our case this was not a reason for not initiating LAI treatment.

Our findings are opposed to the results of other studies. One of them was conducted in Canada, where rates of LAI prescribing, including new starts, discontinuations and switches between LAI types remained highly stable. The authors attributed this mostly to the capacity of the country’s clinics to maintain services for this patient population [8]. Another study, conducted in Pittsburgh, also showed a reduction of only 10% in the LAI injections administered, and only 4 patients were switched to oral antipsychotics at their request [2]. Of note, our study had a longer mirror- image time period (12 months) compared to the other studies, which had a shorter evaluation period- 3 months and respectively 6 weeks. These findings come to show that the impact of COVID-19 pandemic on the entire healthcare system in Romania was probably deeper than originally estimated, and it affected all levels, including hospitals, daycare systems, public and private ambulatory units. This raises questions regarding the management of psychiatric treatments in the eventuality of other pandemics.

**Figure 3:**
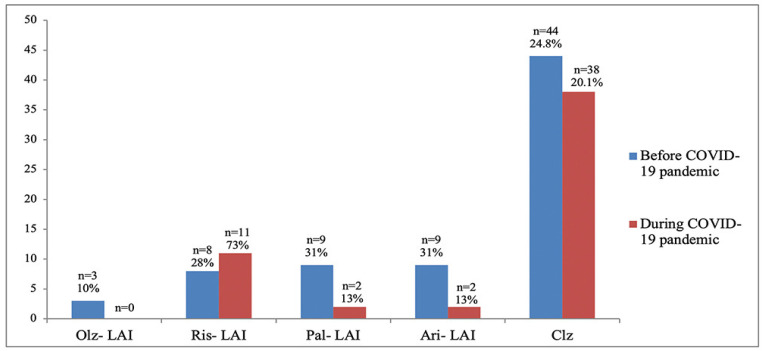
LAIs and clozapine initiations in 2019 vs. 2020. Olz-LAI-olanzapine long acting injectable; Ris-LAI-risperidone long acting injectable; Pal-LAI-paliperidone long acting injectable; Ari-LAI-aripiprazole long acting injectable; Clz-clozapine.

Interestingly, our results showed a minor, non-statistically significant increase in risperidone-LAI initiation, even though this particular LAI has a 2 week administration interval (Figure 3). Based on the administration timetables, it would be reasonable to expect an increase in the initiations of paliperidone-LAI during a pandemic, since this is the only LAI that has a 3-monthly administration interval. This hypothesis was confirmed by one study that found a significant increase in switches from the 1-monthly to the 3-monthly paliperidone during the pandemic [8]. However, our results showed a decrease in the initiation of paliperidone-LAI (9 initiations in 2019 vs. 2 initiations in 2020). A possible reason for this finding could be that the initiation of paliperidone LAI requires 3 administrations in the first 5 weeks.

These findings come in the context of the worldwide underuse of LAIs [15]. Some of the involved factors are related to the patient’s preferences [16,17], to prejudices and stigma, to fear of needles or injection pain [18], to fear of control through medication [19] or to treatment perceived as been coercive [20]. Patients could identify inconveniences (such as the injection frequency, the time loss for travelling to the clinic, high costs) and injectable formulations are generally seen by patients as being related to more severe mental disorders [21,22]. Other factors are related to the clinicians’ limited knowledge and experience, personal beliefs [23], the time spent for convincing the patient and the family [24] (affected in pandemic due to limited interaction recommendation), and to the health systems (availability, cost compensation, etc.) [25,26]. Valuable instruments such as ROLIN (Rating Opportunity for Long-Acting Injectable Antipsychotic Initiation Index) may be a real help for clinicians in their efforts, since it provides a simple and clear picture of the non-adherence history [27].

As per patients’ characteristics, mean age was relatively similar in the two groups of patients (42.3 (±7.8) in 2019 vs. 44.4 (±8.3) in 2020). The mean age in our LAI initiated patients is similar to the results of other studies, and it reflects the real trend in LAI initiation, which in most cases occurs in patients aged over 35 [28]. Patients in the 2020 group were younger at illness onset (25.2 (± 6.6) vs. 28.6 (± 8.6) in 2019) and had a longer duration of illness (19.2 (±6.2) vs. 13.8 (±9.2) in 2019), which could explain the fact that, despite expectations in a pandemic, they had a slightly increased length of stay (16.7 (±6.6) vs. 14.1 (±8.6) in 2019), since they probably required a longer period of time for a good stabilization on an oral treatment before initiation of LAI treatment.

Since we had an interest in the oral antipsychotics on which patients were stabilized, we noticed that the reduction in clozapine initiation after the declaration of COVID-19 pandemic was small and not statistically significant (38 cases in 2020 vs. 44 cases in 2019, *p* = 0.28), despite the fact that this type of treatment requires special monitoring (total blood count, neutrophils, ECG, etc.) and therefore regular visits to health clinics. It would be reasonable to presume that fear of neutropenia associated with clozapine and SARS-CoV-2 infection could determine a replacement or cessation of this treatment [29,30]; however our research found that this did not occur during the pandemic.

## 5. Limitations

There are a number of limitations to our study. First, the number of patients was relatively small, and only one psychiatric center was part of the research. Second, the drop rate for patients already stabilized on LAIs was not considered for this analysis. Lastly, the time period considered for this study was relatively short (12 months before and 12 months after the declaration of COVID-19 pandemic).

The strength of our research resides in the study type: a mirror- image retrospective study.

## 6. Conclusions

The medical landscape has clearly suffered noticeable changes during the pandemic, and some important treatment options for schizophrenia patients were temporarily set aside. However, we must continue to advocate for access to long-acting injectable antipsychotics for schizophrenia patients, even during a pandemic and especially after the introduction of vaccines.

## Figures and Tables

**Table 1:** Antipsychotics with LAI formulations.

Antipsychotic	LAI Formulation	Oral Formulation	Available in Romania
Flupenthixol	√	-	√
Zuclopenthixol	√	-	√
Haloperidol	√	√	√
Fluphenazine	√	√	-
Olanzapine	√	√	√
Risperidone	√	√	√
Aripiprazole	√	√	√
Paliperidone	√	√	√

**Table 2:** LAIs initiation before and during COVID-19 pandemic.

	LAIs Initiation		
	Before COVID-19 Pandemic	During COVID-19 Pandemic	*p* Value
	n = 29	n = 15	
Gender male (n, %)	14 (48.2%)	4 (26.6%)	0.0728
Age (years, SD)	42.3 (7.8)	44.4 (8.3)	0.4121
Age of onset (years, SD)	28.6 (8.6)	25.2 (6.6)	0.1881
Duration of illness (years, SD)	13.8 (9.2)	19.2 (6.2)	0.0476
Length of stay (days, SD)	14.1 (8.6)	16.7 (6.6)	0.3120
LAI type			
aripiprazole (n, %)	9 (31%)	2 (13%)	0.1955
olanzapine (n, %)	3 (10%)	0	-
risperidone (n, %)	8 (28%)	11 (73%)	0.2036
paliperidone (n, %)	9 (31%)	2 (13%)	0.1955

SD-standard deviation.

**Table 3:** Oral antipsychotics.

	2019	2020	*p* Value
OAs (n, %)	177 (79%)	189 (86.7%)	0.0322
olanzapine (n, %)	61 (34.5%)	69 (36.5%)	0.6899
quetiapine (n, %)	18 (10.2%)	24 (12.7%)	0.4542
risperidone (n, %)	18 (10.2%)	23 (12.2%)	0.5454
paliperidone (n, %)	3 (1.7%)	7 (3.7%)	0.2415
aripiprazole (n, %)	8 (4.5%)	6 (3.2%)	0.5178
amisulpride (n, %)	14 (7.9%)	10 (5.3%)	0.3160
haloperidol (n, %)	11 (6.2%)	12 (6.3%)	0.9685
clozapine (n, %)	44 (24.8%)	38 (20.1%)	0.2816

## Data Availability

Data was retrieved from the paper and electronic documents of the patients. The datasets used and/or analyzed during the current study are available from the corresponding author upon reasonable request.

## Abbreviations

LAI	long acting injectable
COVID-19	coronavirus disease 2019
SARS-CoV-2	severe acute respiratory syndrome coronavirus 2
FGA	first generation antipsychotics
SGA	second generation antipsychotic
WHO	World Health Organization
OA	oral antipsychotic
Olz-LAI	olanzapine long acting injectable
Ris-LAI	risperidone long acting injectable
Pal-LAI	paliperidone long acting injectable
Ari-LAI	aripiprazole long acting injectable
Clz	clozapine
